# Nuclear matrix protein 2 antibody-positive dermatomyositis associated with hepatocellular carcinoma

**DOI:** 10.1093/rap/rkad066

**Published:** 2023-08-01

**Authors:** Yoshitaka Ueda, Kota Shimada

**Affiliations:** Department of Rheumatic Diseases, Tokyo Metropolitan Tama Medical Center, Fuchu, Tokyo, Japan; Department of Rheumatic Diseases, Tokyo Metropolitan Tama Medical Center, Fuchu, Tokyo, Japan

Key message

- Adult patients with anti-NXP-2 antibody-positive dermatomyositis may be associated with hepatocellular carcinoma.

Dear Editor, DM is a systemic idiopathic inflammatory myopathy characterized by proximal skeletal muscle weakness and chronic inflammation of the skin and muscle. Nuclear matrix protein 2 (NXP-2) is involved in the regulation of p53-induced cellular senescence in response to oncogenic signals. The anti-NXP-2 antibody is myositis specific and more common in younger, Caucasian patients [1]. The antibody is present in 20% of JDM cases [2] but is less common in adult DM, where it is known to be associated with malignancies [3]. Although the association between DM and malignancies is well known, DM is not widely recognized as a paraneoplastic syndrome of hepatocellular carcinoma (HCC). Herein, we present an adult patient with anti-NXP-2 antibody-positive DM associated with HCC.

A 59-year-old male patient presented at the previous treatment centre with fatigue of 1 week in duration. Abdominal CT with contrast medium revealed a liver tumour, and the patient was referred to the Department of Gastroenterology at the study centre. The initial laboratory data revealed elevated enzymes, with aspartate aminotransferase (AST) 267 U/l, alanine aminotransferase (ALT) 101 U/l, alkaline phosphatase (ALP) 130 U/l, γ-glutamyltransferase (γ-GT) 422 U/l, lactate dehydrogenase (LDH) 646 U/l and creatine kinase (CK) 3198 U/l. The carcinoembryonic antigen and carbohydrate antigen 19-9 titres were almost normal, and elevated levels of α-fetoprotein (19 863 ng/ml) and proteins induced by vitamin K absence-II (62 133 mAU) were observed. HBsAg, antibody to HBsAg, hepatitis B core protein and anti-hepatitis C antibody were negative. Liver dynamic CT revealed a 10 cm, arterially enhancing mass in hepatic segment 4, consistent with HCC (Fig. 1A). Based on these findings, HCC stage T4N0M0 was diagnosed, and after 1 week he was admitted to the Gastroenterology Department for treatment. On admission day, a significant decrease in muscle strength in the extremities was observed, and the patient was referred to the Department of Rheumatic Diseases for further testing. Two weeks before hospitalization, he had difficulty lifting objects. His muscle weakness progressed, and difficulty in swallowing occurred several days before hospitalization. He had no history of fever, dyspnoea, cough or myalgia. His medical history included diabetes mellitus and hypercholesterolaemia. His current medical prescriptions included metformin, dapagliflozin, linagliptin and pravastatin. He had a 10-year history of smoking and drank socially. He had no known family history of rheumatic diseases. A physical examination revealed multifocal pruritic, erythematous plaques on the extremities and trunk (Fig. 1B), and no skin lesions typically associated with DM, such as heliotrope rash or Gottron’s sign, were observed. A musculoskeletal examination found grade 3–4/5 muscle strength in the symmetrically proximal and distal muscles of the bilateral upper extremities. Autoantibodies, including anti-SSA, anti-SSB, anti-RNP, anti-Sm, anti-dsDNA, anti-Jo-1, anti-PL-7, anti-PL-12, anti-EJ, anti-OJ, anti-KS, anti-Mi2, anti-SAE and anti-TIF1-gamma were negative, but anti-NXP-2 antibody was positive. MRI of both lower extremities revealed areas of high signal intensity in the bilateral thigh muscles. No skin or muscle biopsy was performed. HCC-associated DM was diagnosed on the basis of these findings. Pravastatin was discontinued, and treatment with prednisolone, tacrolimus and IVIG was begun. Transcatheter arterial chemoembolization for HCC was also begun, and surgical treatment and proton beam therapy were considered but eventually ruled out because of the patient’s poor liver function. Although complete restoration of muscle strength was not achieved, some therapeutic response was observed through immunosuppressive therapy, and the patient was able to be discharged.

**Figure 1.**
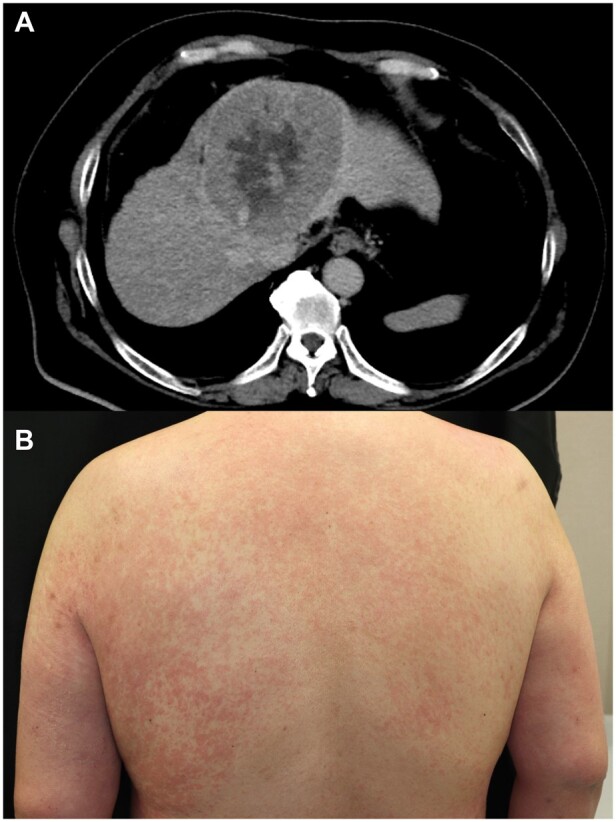
CT image of hepatocellular carcinoma and clinical image of skin lesions. (**A**) Contrast-enhanced CT revealed a tumour with a long axis of 10 cm in the S4 segment of the liver. Necrotic changes were visible in the central region. (**B**) Extensive coalescing oedematous erythema was observed on the limbs and trunk

A previous study reported that 1.6–3.0% of cases of adult myositis were positive for the anti-NXP-2 antibody [4], which is associated with calcinosis, s.c. oedema and dysphagia [1]. Moreover, adult patients with anti-NXP-2 antibody-positive DM are often associated with malignancies [3]. To the best of our knowledge, there are no reports of anti-NXP-2 antibody-positive DM associated with HCC. Although there are some reports of HCC-associated DM [5, 6], they do not include anti-NXP-2 antibody-positive cases, and it is speculated that the limited availability of facilities capable of easily measuring the antibody is one contributing factor. The detection rate is lower for this antibody than for the anti-TIF1-gamma antibody, which is frequently detected in cases of cancer-associated DM [7]. Nonetheless, whenever a patient presents with cancer-associated DM, testing for anti-NXP-2 antibody should be done if possible. Conversely, if an adult patient has DM with anti-NXP-2 antibody positivity, an examination for malignancies is necessary.

DM associated with HCC is rare [8], and many patients with this condition die <1 year after DM diagnosis [5]. Large tumours are often found in cases of DM associated with HCC and are believed to affect the prognosis. Large HCC lesions often accompany reduced liver function, making it less amenable to surgical resection or radiation therapy and therefore more strongly associated with a poor prognosis. In the present case as well, curative treatment for the HCC was challenging.

## Data Availability

Derived data supporting the findings of this case report are available from the corresponding author on request.
